# Detecting the Severity of Socio-Spatial Conflicts Involving Wild Boars in the City Using Social Media Data

**DOI:** 10.3390/s21248215

**Published:** 2021-12-08

**Authors:** Małgorzata Dudzińska, Agnieszka Dawidowicz

**Affiliations:** Institute of Geography and Land Management, Faculty of Geoengineering, University of Warmia and Mazury in Olsztyn, 10-720 Olsztyn, Poland; gosiadudzi@uwm.edu.pl

**Keywords:** wildlife, multimedia data, photointerpretation, spatial analysis, GIS, socio-economic geography

## Abstract

The encroachment of wild boars into urban areas is a growing problem. The occurrence of wild boars in cities leads to conflict situations. Socio-spatial conflicts can escalate to a varied degree. Assessments of these conflicts can be performed by analyzing spatial data concerning the affected locations and wild boar behaviors. The collection of spatial data is a laborious and costly process that requires access to urban surveillance systems, in addition to regular analyses of intervention reports. A supporting method for assessing the risk of wild boar encroachment and socio-spatial conflict in cities was proposed in the present study. The developed approach relies on big data, namely, multimedia and descriptive data that are on social media. The proposed method was tested in the city of Olsztyn in Poland. The main aim of this study was to evaluate the applicability of data crowdsourced from a popular social networking site for determining the location and severity of conflicts. A photointerpretation method and the kernel density estimation (KDE) tool implemented in ArcGIS Desktop 10.7.1 software were applied in the study. The proposed approach fills a gap in the application of crowdsourcing data to identify types of socio-spatial conflicts involving wild boars in urban areas. Validation of the results with reports of calls to intervention services showed the high coverage of this approach and thus the usefulness of crowdsourcing data.

## 1. Introduction

Human encroachment into wildlife habitats, including into areas colonized by wild boars, has contributed to the presence of wild animals in cities. Each year, these problems are exacerbated not only by progressing urbanization, where wild boar habitats are lost as cities expand their administrative boundaries, but also by development projects that intersect wildlife migration corridors [1,2,3]. The rapid growth of the European population of wild boars (*Sus scrofa*) in the past decade has also contributed to the above problem [3,4,5]. Wild boars have quickly adapted to life in the urban environment, which is a source of food and shelter, and where the wild boar population increases rapidly due to the absence of other large predators [6,7]. A preliminary analysis of data from the intervention reports generated by the City Guard in Olsztyn in north-eastern Poland revealed that the presence of wild boars in Olsztyn and the number of City Guard interventions to protect local residents and property increased during the COVID-19 pandemic when social mobility was restricted.

The encroachment of wild boars into urban areas poses a threat for both animals and humans. The occurrence of wild boars in cities can violate public order and compromise the residents’ safety (damage to property and production facilities, traffic disruptions and, in extreme cases, attacks on humans and pets, and transmission of disease) [8,9]. In many cases, the presence of wild boars in urban areas can lead to conflict. According to [8], “human-wildlife conflicts are caused where the movement and activities of wildlife, such as associated with foraging or reproduction, have an adverse impact on human interests, such as through aggression or nuisance behavior”.

Socio-spatial conflicts can escalate to a varied degree, and they require different interventions and solutions. Therefore, the type and severity of human–wild boar conflicts in urban areas should be accurately defined to select the most appropriate remedy measures. Such assessments can be performed by analyzing spatial data concerning the affected locations and wild boar behaviors, in particular human–wild boar interactions in urban space. The collection of spatial data is a laborious and costly process that requires access to urban surveillance systems, in addition to regular analyses of intervention reports. This is an effective approach, but does not always generate the necessary information [10,11]. A preliminary study revealed that not all wild boar “incidents”, in the sense of seeing the boar from a distance and close contact (interaction) with the boar are reported to municipal security services. The above may indicate that local communities have become accustomed to the presence of wild boars or that the perceived threat was low. Public services that respond to wild boar incidents, including the police, the fire department, and the city guard, keep separate databases, but if the accumulated data are incomplete, these services may respond too late and the applied remedy measures may be inadequate. Therefore, additional supporting methods of accumulating important data about incidents of human–wildlife interactions are needed [11,12]. Wildlife enthusiasts who share the relevant information online on a crowdsourcing basis are a valuable source of such data [13]. Data can also be crowdsourced on social media platforms, where users generate geographic data, including information about the location of various phenomena.

In view of the above, a supporting method that enables the extension of information for evaluating the threats associated with the presence of wild boars and socio-spatial conflicts in cities was proposed in the present study. The developed approach relies on big data, namely, multimedia and descriptive data that were generated and posted by the residents of the Polish city of Olsztyn on social media. The proposed method was designed to test the applicability of crowdsourced data for evaluating the severity of socio-spatial conflicts. The presented approach is cost- and time-effective because it involves only processing and interpretation of crowdsourced data (no on-site inventory is needed). It does not require an investment of financial means to produce the data. This finding is confirmed by the research of [14], where “reports of wild pig sightings from community members remain a cost-effective tool to detect low-density invasive species across large regions”. The described method is not burdensome for content creators, namely, a crowd of wildlife enthusiasts who are active online, and it does not compromise the quality of the generated multimedia data. Unlike common mapping techniques, the proposed method does not require expert knowledge to develop and publish spatial data [15]. The described approach is a passive method, and unlike active data collection campaigns, it can capture differences in opinions and attitudes in space and time [16]. Unprocessed and unmodelled data generate the most reliable results.

Therefore, the main aim of this study was to evaluate the applicability of crowdsourced data from a popular social networking site (“Wild boars in Olsztyn”, started by a local group of wildlife enthusiasts) for determining the location and severity of human–wild boar conflicts in urban space. A photointerpretation method and the kernel density estimation (KDE) tool implemented in ArcGIS Desktop 10.7.1 software were applied in the study. A review of the literature revealed that a classification system for assessing socio-spatial conflicts involving wildlife in urban areas has not been proposed to date. Therefore, a detailed goal of this study was to develop a classification of human–wild boar conflicts in the city based on own research data and an analysis of the literature.

The results of a preliminary analysis of spatial data were used to formulate the following research hypothesis: multimedia data generated by local community members can support the process of identifying socio-spatial conflicts involving wild boars for the determination of the location, severity, and distribution of wild boars in urban areas. The proposed approach can be a useful, supporting tool for observing and assessing human–wildlife conflicts, and it can be used by local authorities to develop fit-for-purpose solutions to alleviate socio-spatial conflicts between humans and wild boars and to implement remedy measures.

This important research direction is driven by the opportunities and advantages of the information society, where information is widely shared with the use of digital technologies. Digital inclusion promotes involvement in social initiatives on an unprecedented scale. Social activists rely on social media to organize protests and events, particularly in large cities [17]. The relevant data can be a valuable and up-to-date source of information about human activities and interactions with the natural environment in various spatial and temporal scales [16]. A review of the literature indicates that crowdsourced social media data have been used to map cultural ecosystem services [18], evaluate visitors’ preferences regarding biological diversity [19], monitor biological diversity [11], monitor visitors in nature conservation areas, monitor social responses to environmental protection events [20,21], explore global trends in wildlife trade [22], and monitor of animal movements [14,23]. These models lead to an assessment of relevant quantities, such as the probability of animals entering/escaping from an urban area, in addition to typical traffic patterns, which in turn lead to an estimate of the risk of human–wildlife interactions [24,25,26,27]. The proposed approach, together with the parallel studies of [14], fill a gap in the application of crowdsourced data to identification of incidents involving wild boars in urban areas. The proposed method additionally identifies the types of socio-spatial conflicts involving wild boars. Therefore, the proposed approach testifies to the innovative character of this study.

## 2. Materials and Methods

### 2.1. Methodology

**Figure 1:**
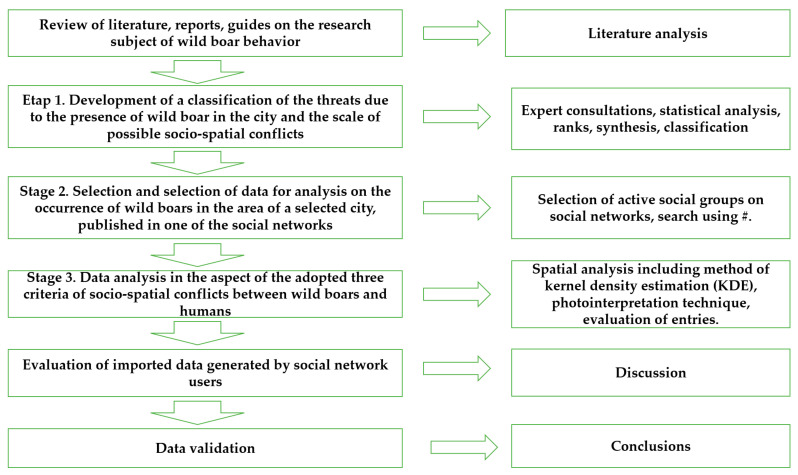
Diagram of research methods and tasks. Source: the authors.

The research methodology involved analytical methods, including analyses of scientific literature, reports, guidebooks relating to wild boars, multimedia, and descriptive data. The results of these analyses were spatially processed. The authors conducted a quantitative empirical study (distribution of human–wild boar conflicts in the city, evaluation of wildlife enthusiasts who create online content) and a qualitative study (photointerpretation of multimedia files, and determination of the severity and distribution of human-wild boar conflicts in the city). The research methods and the performed research tasks are presented in Figure 1.

The sequence of the planned research tasks was set based on the adopted methodological assumptions to determine the applicability of crowdsourced data and to validate the research hypothesis, postulating that multimedia data generated by local community members can support the process of identifying socio-spatial conflicts involving wild boars for the determination of the location, severity, and distribution of wild boar in urban areas.

In the first stage of the study, potential socio-spatial conflicts involving wild boars were classified, and a list of possible threats was developed based on a review of the literature, City Guard reports, a report of the Supreme Audit Office, and thematic guidebooks. The list of threats was assessed by independent experts, and the results were used to develop a scale for measuring the severity of different types of conflict. The expert group comprised professionals and scientists with extensive knowledge and experience in forestry, environmental protection, psychology, and damage assessment (property appraisers). In the next stage of the study, posts containing information about the occurrence of wild boars in the city were searched on a popular social networking site, using “wild boar” and “Olsztyn” as the key search terms, preceded by the hash symbol #. Posts containing such information were selected for analysis.

In the last stage of the study, the data selected for analysis were processed and visualized to determine the severity of socio-spatial conflicts involving wild boars in Olsztyn. Historical and descriptive data published on the examined social networking site were analyzed statistically, and the attached multimedia files concerning the city of Olsztyn in north-eastern Poland were interpreted. The results of spatial analyses were presented on cadastral maps with the use of ArcGIS software. Spatial analyses were conducted with GIS tools and the KDE method. The applicability of crowdsourced social media content for generating information about human–wild boar conflicts was discussed. The severity of human–wild boar conflicts in urban areas varies over time; therefore, the study covered a period of eight months as the minimum observation period that accounts for the mating season (wild boars have a gestation period of three months). The urban environment is an abundant and steady source of food, and wild boars can breed up to three times per year in cities [28]. Therefore, the eight-month study period supported observations of all types of human–wild boar conflicts during the entire breeding cycle.

The results were validated by comparing them with the numbers of security forces’ interventions into wild boars and their localization from reports of calls during the same time period.

### 2.2. Study Area

The study involved an analysis of crowdsourced social media data concerning the presence of wild boars in Olsztyn. The city was selected for the study because the authors live in Olsztyn, which enabled them to verify the locations where wild boars had been sighted by social media users. Olsztyn was also chosen due to its location and unique environment. Olsztyn is the capital city of Warmia and Mazury, a region with the highest number of nature reserves in Poland, and is visited by many species of wild animals.

**Figure 2:**
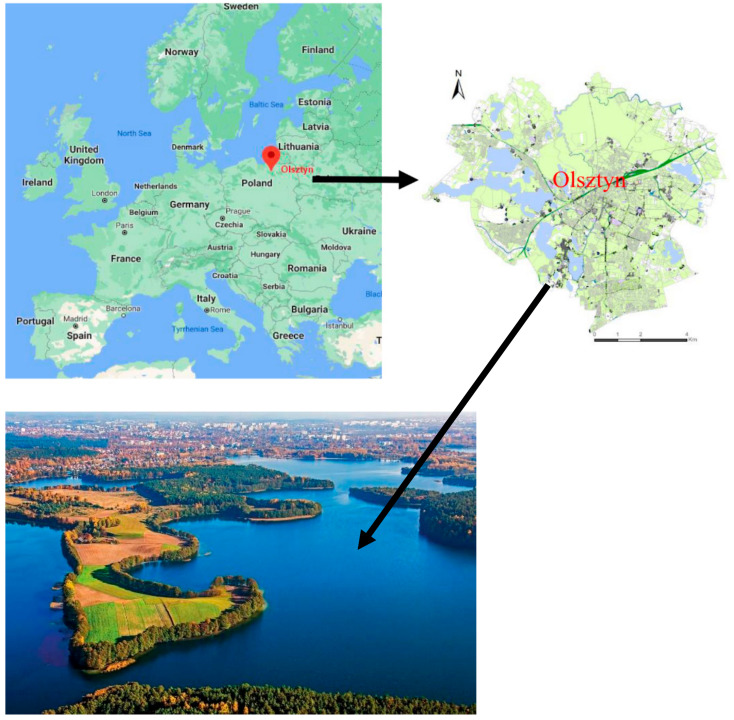
Location of the city of Olsztyn. Source: own elaboration.

Olsztyn is located on the Łyna River in north-eastern Poland, in the proximity of the borders with Lithuania, Belarus, and Russia (Kaliningrad Region) (Figure 2). It is the capital city of the Region of Warmia and Mazury, which is known as the “Land of a Thousand Lakes” (there are 15 lakes within the city’s administrative boundaries that occupy 10% of Olsztyn’s area) and the “Green Lungs of Poland” (with the highest forest cover in Poland). Both Olsztyn and the Region of Warmia and Mazury are unique in terms of biological diversity and species richness. The city occupies an area of 88.33 km^2^ and has a population of 172,000.

## 3. Results

### 3.1. Definition of Socio-Spatial Conflict between Humans and Wild Boars

A review of the literature on wild boars and their occurrence in cities revealed that the risks associated with the presence of wild boars in urban areas and the resulting socio-spatial conflicts showed a small number of studies, especially focused on the identification of incidents involving wild boars. Published studies contain only fragmentary descriptions of incidents involving wild boars, and the frequency and severity of wild boar encroachment into human areas has not been evaluated on a dedicated scale. Therefore, the concept of socio-spatial conflict was used to describe the scale of incidents (interactions) between humans and wild boars in urban space. To fill the existing knowledge gap, a list of possible risks associated with the presence of wild boars in cities was developed based on an analysis of the literature. These risks were classified in the context of: the wild boar biology [5] ecology approach, wild boar activity in cities [7,29,30], human–wild boar interactions in cities [8,9], intervention reports filed by municipal security services, preliminary analyses of social media posts, and the authors’ own observations.

Wild boars (*Sus scrofa*) belong to the family *Suidae* and are classified as game animals in the Polish legal system [31]. Wild boars are considered large game animals due to their size. Adults have a length of 1–1.5 m and weigh around 200 kg (males can weigh as much as 300 kg). Wild boars resemble domesticated pigs, but their bodies are covered with dark, coarse hair, and they have much stronger tusks that protrude from the mouth.

**Figure 3:**
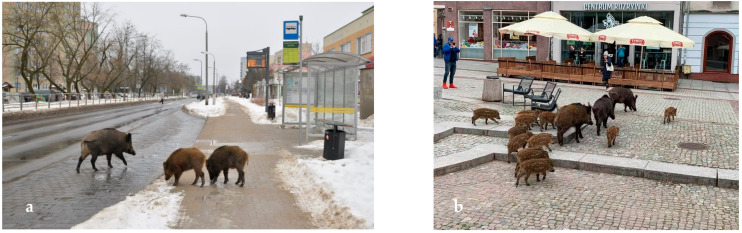
A wild boar in Olsztyn’s urban environment ((**a**) Kormoran housing estate in Olsztyn, (**b**) Old Town in Olsztyn). Source: R. Borawski and H. Sitek.

Wild boars live in large family groups that consist of up to 20 individuals, including females and their offspring. Adult males are usually solitary, and they join groups only during the breeding season, which lasts from November to January. During that period, males fight ferociously for access to females. In wild boars, pregnancy lasts nearly three months, and the average litter consists of four to eight piglets [9]. Wild boars command respect and are feared due to their strength, appearance, and biology. Their innate behaviors pose a danger not only to wild boar groups, where they fight to establish dominance and protect their young, but also to other species and populations, including organized human communities in the urban environment. Wild boars damage property, disrupt road traffic, and are feared by city dwellers (Figure 3).

For these reasons, human–wild boar conflicts in cities and their severity should be identified. A list of various types of socio-spatial conflicts involving wild boars was developed. Four categories and 10 possible types of conflicts were included on the list:

(1) Damage to property: (I) resulting from foraging behavior (for example, for scarab beetles) which causes damage to lawns, flower beds, gardens, and parks; (II) resulting from nesting behavior which causes damage to lawns, flower beds, and shrubs; (III) public sanitation hazard (wild boars forage for food by overturning garbage cans and leaving garbage strewn over pavements and streets);
(2) Safety risk (health risk): (IV) wild boars cross public roads and forage on road lanes; (V) wild boars cause traffic accidents; (VI) wild boars attack humans;
(3) Psychological risks (fear and anxiety) associated with: (VII) presence of wild boars near adults; (VIII) presence of wild boars near children and safe child zones such as playgrounds; (IX) fighting between male wild boars, copulation, sows protecting their offspring (risk of attack on humans);
(4) Secondary risks: (X) transmission of parasites and disease.

Twelve independent experts who have extensive knowledge of wild boar behaviors and adaption to city life, in addition to professional experience in dealing with wild animals in cities, were surveyed, and the obtained information was used to rank the identified categories and types of socio-spatial conflicts and determine their severity in the studied city. The panel of experts consisted of three forest managers, three property appraisers, three environmental protection experts, and three psychologists. The experts were asked to complete a questionnaire. The questionnaire was anonymous as not to put pressure on the experts, and the surveyed subjects were only requested to indicate their field of expertise. The selection of three independent experts from each area of expertise made it possible to verify the comparability of results within these areas. Therefore, in the first three questions the respondents were asked to state their age, professional experience, and field of expertise. In the following question, the experts were asked to rank (ascribe weights to) the listed threats associated with the presence of wild boars in the city, and to propose other types of risks which were not included in the list. The severity of the identified threats was graded on a four-point scale, where a higher score represented a more serious risk: high risk—3 points, moderate risk—2 points, low risk—1 point, no risk—0 points. The respondents were then asked to indicate the extent to which the occurrence of wild boars could pose a threat in various locations in city. The experts were presented with four locations (peripheral areas, residential districts, downtown Olsztyn, and other locations). The severity of the threat in each location was evaluated on a three-degree scale (low, moderate, high) (Table 1). The last question concerned the nuisance associated with the presence of wild boars in Olsztyn. In some cases, wild animals encroach into cities without posing a threat or causing damage. Therefore, the experts were asked to indicate situations in which the presence of wild boars is a public nuisance. It was assumed that such an assessment can be made based on the number of threats reported in the city in a given period of time, such as a month. The experts were asked to state the number of wild boar threats that is indicative of low, moderate, and severe nuisance. The results were grouped in intervals. The answers to the last two questions are presented in Table 1.

The results of expert questionnaires were processed statistically in the next stage of the study. It was assumed that all answers were significant because they were provided by experts in the field. Therefore, to determine the severity of wild boar threats and to visualize the distribution of wild boar incidents on a map, the weights for each type of socio-spatial conflict between humans and wild boars were calculated with the use of a weighted median for the cumulative sum of values. The median is least sensitive to outliers, and in this case, it supported reliable predictions of the severity of specific threats. The results of the statistical analysis are presented in Table 2.

The statistical analysis revealed a high degree of agreement between the respondents. All of the identified threats were regarded as significant and received 1 to 3 points on the grading scale. Conflicts that pose a direct threat to human safety were ranked highest. These types of threats included traffic accidents caused by the presence of wild boars on public roads or in their proximity, in addition to attacks on humans. The presence of wild boars in the proximity of children and extensive property damage (flower beds, gardens, shrubs, and parks) in the city also received the highest scores. According to the surveyed experts, the resulting nuisance can be classified as severe if more than 20 threats associated with the occurrence of wild boars are reported per month. The presence of wild boars in downtown Olsztyn was also regarded as a severe nuisance.

Based on the results of the questionnaire survey, the authors decided that the severity of socio-spatial conflicts involving wild boars in the city should be determined by evaluating: type of threat (Table 2), location of threat (locations where wild boars were sighted) (Table 1), and the frequency of wild boar occurrence in the city (Table 1). A scale was developed for assessing the severity of socio-spatial conflicts involving wild boars in urban areas. Three degrees of threat intensity were identified based on the significance of the above three factors:

1st degree threat: Low—Location of threats in urban space—1 point, frequency of threat—1 point (up to 10 threats per month), type of threat—Mostly 1 or 2 points;

2nd degree threat: Moderate—Location of threats in urban space—2 points, frequency of threat—2 points (11–20 threats per month), type of threat—1 or 2 points, sporadically 3 points;

3rd degree threat: High—Location of threats in urban space—3 points, frequency of threat—3 points (more than 20 threats per month), type of threat—1, 2 or 3 points.

### 3.2. Analysis of Crowdsourced Data from Social Media

Data were crowdsourced from a popular social networking site containing posts on the occurrence of wild boars in Olsztyn. Metadata were collected from the interface, and the search endpoint was set at 30 October 2020. A preliminary analysis of selected data revealed that the evaluated social networking site contained not only individual posts on the occurrence of wild boars in the city, but also packages of data collected by a group of wild boar enthusiasts. One private group (“Wild boars in Olsztyn”) and three websites aggregating information about wild boars in Olsztyn (“Olsztyn loves wild boars”; “Where are wild boars Olsztyn”; and “Wild boars in Olsztyn”) were identified. The number of dedicated social groups and websites indicates that the occurrence of wild boars in the city is a serious and current problem. These findings confirm that social networks can be a supportive “detector” of various phenomena in the urban space, including threats [32,33]. Detailed information about crowdsourced data concerning wild boars in Olsztyn is presented in Table 3.

The “Wild boars in Olsztyn” group generated the largest volume of information and the most detailed data. The group aggregates multimedia data, including photographs and video footage posted by users. A share of 99% of the collected multimedia files contain photographs of wild boars in the city. These resources were filtered by eliminating files that did not contain images of wild boars in Olsztyn. To ensure that the results of the analysis were not distorted by content creators, who tend to publish numerous photographs from a single sighting, the number of photographs linked to a given post was limited to five photographs per person. A total of 606 multimedia files (photographs and video footage) were analyzed. Photographs and posts were evaluated in the context of socio-spatial conflicts involving wild boars with the use of the developed classification scale (Table 1 and Table 2). The geographic location of the key interactions between humans and wild boars was determined based on the digital footprint [34] of the users’ mobile devices and their social media activity, in the spatial and temporal resolution of geotagged images. The preferences and characteristics of content creators were also analyzed, including sex, age, socioeconomic status, and potential motivation [35,36,37,38]. The majority of active users were Olsztyn residents (84%) and persons temporarily residing in the city (school and university students, employees). An analysis of user profiles revealed that content creators belonged to various age groups, ranging from adolescents (students) to working adults of both sexes. All active users had an interest in wildlife and were enthusiastic about spotting wild boars in urban space.

**Figure 4:**
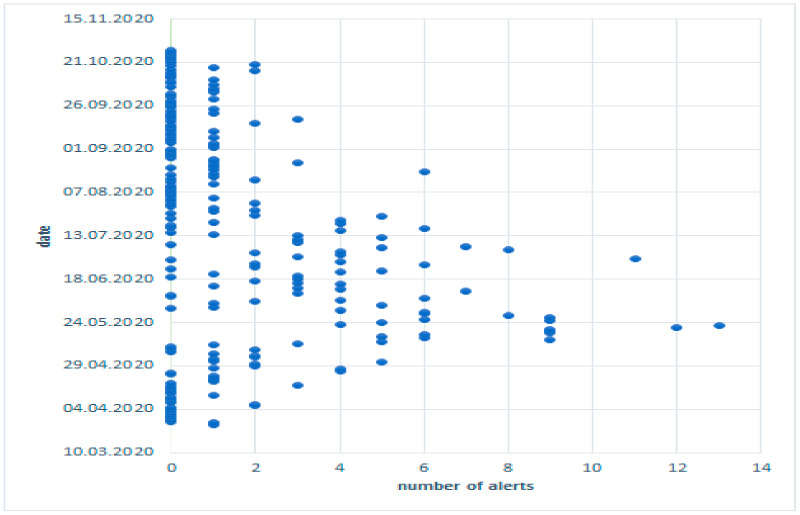
Frequency of posts in the “Wild boars in Olsztyn” group. Source: the authors.

**Figure 5:**
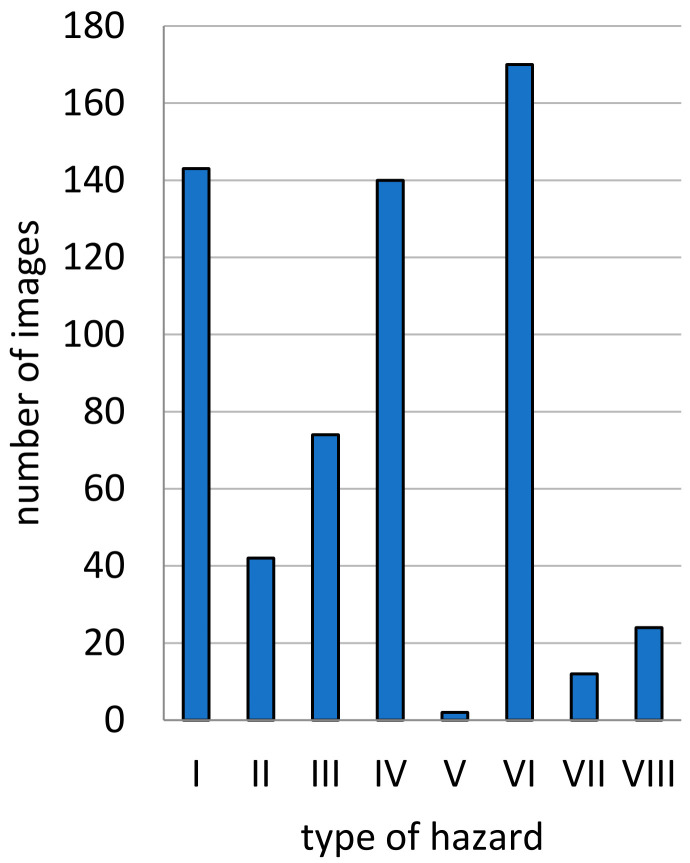
Frequency of various types of threats associated with the occurrence of wild boars in the city of Olsztyn. Source: own elaboration.

The frequency of posts related to the occurrence of wild boars in Olsztyn (Figure 4) and the number of the resulting threats were analyzed. The results were regarded as significant because 401 posts were published and 606 threats were identified (Figure 5).

### 3.3. Interpretation of Crowsourcing Data

In the analyzed period, wild boars were sighted up to several times a day in different parts of Olsztyn (Figure 5). A record-breaking number of incidents (13) was reported on 24 May. However, the occurrence of wild boars is not always associated with the classified threats and types of damage. In some cases, the animals encroached into various parts of the city, but kept their distance from humans and did not cause any dangerous incidents. Therefore, attempts were made to identify situations in which wild boars posed a threat.

The number and types of threats associated with the occurrence of wild boars in Olsztyn were determined with the use of the photointerpretation method. Photographs and video footage were evaluated, and the results were compared with a catalogue of previously observed and identified spatial conflicts between humans and wild boars (Table 1 and Table 2). In recent years, photointerpretation has emerged as an important research method, and has been used in various types of studies [39].

**Figure 6:**
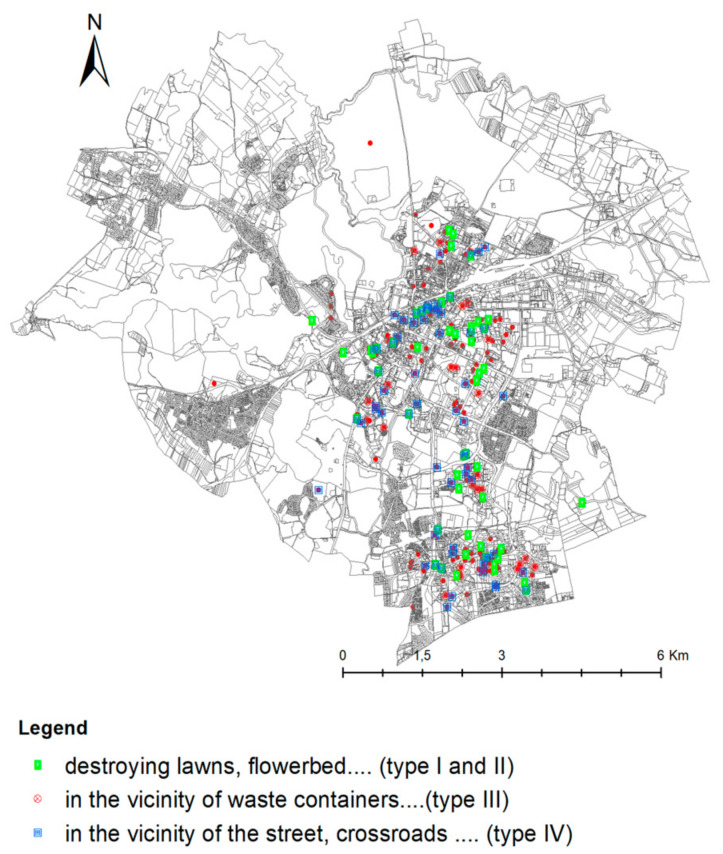
A map presenting the spatial distribution of threats in the city of Olsztyn. Source: own elaboration.

Every selected multimedia file was analyzed individually for the presence of the classified threats. The number of threats identified by photointerpretation in the examined multimedia files (606 files) is presented in Figure 5, and their location is shown in Figure 6. Many multimedia files (photographs and video footage) depicted several types of threats.

**Figure 7:**
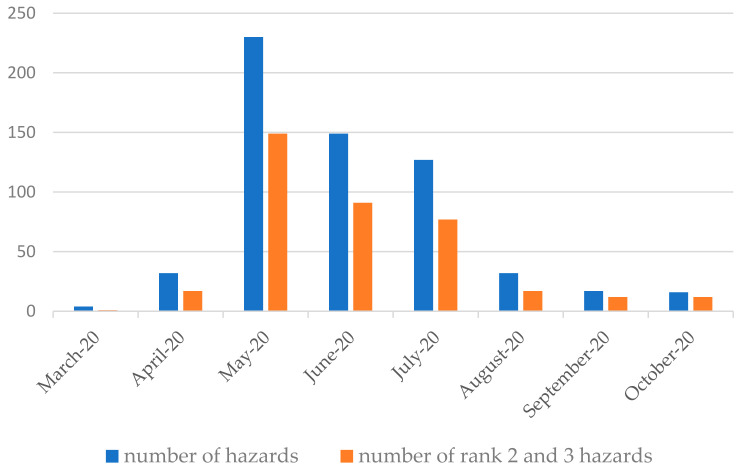
Monthly number of threats associated with the presence of wild boars in the city of Olsztyn. Source: the authors.

More than 600 wild boar threats were identified in the city, and nearly ⅓ of those incidents were classified as moderate and severe (Figure 7). The number of socio-spatial conflicts in Olsztyn was highest in May and June 2020, when 235 and 149 incidents were reported, respectively. The number of threats continued to decrease in the following months, and reached 17 in September and 16 in October.

#### 3.3.1. Localization of Wild Boar Incidents in the City of Olsztyn

**Figure 8:**
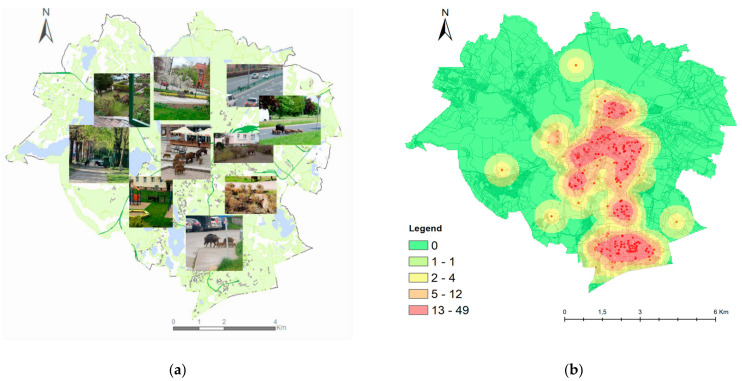
The maps depicting wild boar activity in the city of Olsztyn between March and October 2020 ((**a**) type of wild boars’ activities; (**b**) wild boars’ concentration in the city). Source: own elaboration.

The locations where wild boars were sighted in Olsztyn were determined by analyzing the information provided by active users (post authors) and by interpreting geographic identification metadata in geotagged images (street names, characteristic urban sites, street junctions, squares, shops, buildings, etc.). The information provided by content creators was verified. Google Street View maps were used to identify 212 locations where wild boars were sighted in Olsztyn. The identified sites and the geographic distribution (density) of wild boar incidents (per km^2^) are presented in Figure 8. A spatial analysis was conducted in the ArcGIS Desktop 10.7.1 program with the use of GIS tools and the KDE method. The non-parametric KDE method was applied to determine the frequency of wild boar occurrence in the city. This interpolation method is used to transform data from point-based observations into continuous fields. Kernel density estimation is widely used in spatial analyses to convert sets of geographically dispersed data to dense clusters in the GIS environment [40].

The highest number of wild boar incidents were in the central and southern parts of city which mainly feature residential districts (Jaroty, Nagórki, Pieczewo, Zacisze), and in downtown Olsztyn. These areas are characterized by high population density and high traffic, but they also feature urban green spaces and municipal parks. The locations that were most frequently visited by wild boars in the analyzed months were also identified. This information is important because it can be used to predict wild boar behaviors in the future. Wild boars were sighted in the residential district of Jaroty in March 2020. Between April and August 2020, the animals were spotted not only in residential districts and peripheral areas, but also in the city center. In September and October 2020, wild boars were reported in residential districts, peripheral areas and, sporadically, in downtown Olsztyn.

The results of the photointerpretation indicate that wild boars are not afraid of humans. Nearly half of the evaluated photographs were taken in residential districts, parks, and even the Old Town. In some photographs, wild boars were captured in the vicinity of large supermarkets (Lidl, Biedronka). The destruction wrought by wild boars was depicted in 108 photographs. The animals caused extensive damage to lawns, flower beds, public parks, sports facilities, and the municipal cemetery, and generated significant economic losses in the city center. Wild boars also caused traffic disruptions by crossing public roads and foraging in the vicinity (99 photographs). Two video recordings presented traffic accidents involving wild boars. Numerous multimedia files depicted wild boars foraging in landfills (74 photographs). The animals scattered trash in the vicinity of foraging sites. Police interventions aiming to repel wild boars were presented in only five multimedia files.

An analysis of the photographs leads to the conclusion that wild boards regard urban areas as their second home because they roam freely in cities and meet their physiological needs without restraint: males fight for dominance and access to females; wild boars copulate; sows feed their offspring on lawns; and the animals rest and build nests in the city (in particular in shaded areas under balconies in residential estates or under trees in parks). Wild boars invade cities not only in search of food. Due to regular and prolonged contact with humans, wild boars have grown accustomed to the urban environment, and their behavior has also changed in non-urban habitats [41].

The analysis of multimedia files and user posts also revealed that some individuals (mostly young people) feed wild boars, try to pet them and encourage their children to do so, and accept the presence of wild boars in residential districts and the city center. Several people have attempted to attract wild boars’ attention by calling them. Children regard wild boars as an attraction. Some residents, in particular young people, ignore the fact that boars are wild animals.

#### 3.3.2. The Scale of Intensity of the Phenomenon of Socio-Spatial Conflict in the City of Olsztyn

The results of the photointerpretation of multimedia files (Table 4) were used to determine the intensity of the observed phenomenon, i.e., to rank the severity of socio-spatial conflicts involving wild boars in the city of Olsztyn.

The severity of the evaluated conflicts varied over time. The number of wild boar incidents was highest between April and August 2020, and it was moderate in September and October 2020. The analysis did not cover all of March, which is why the severity of human–wild boar conflicts was evaluated as low in that month. Two online communities publishing information about wild boars in Olsztyn were established in March 2020: a private group called “Wild boars in Olsztyn” and a website having the same name (Table 3).

#### 3.3.3. Data Validation

**Figure 9:**
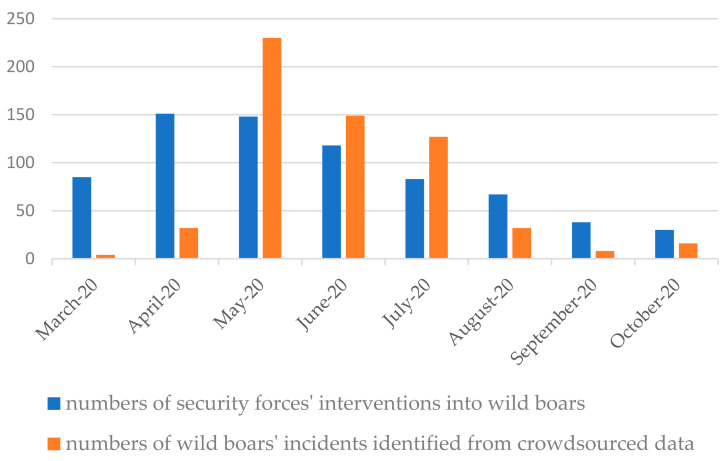
Comparison of the number of wild boar incidents from security forces’ interventions reports and social media data. Source: own study.

The results were validated by comparing them with the numbers of security forces’ interventions into wild boars and their localization from reports of calls during the same time period (March–October 2020.). During the analyzed period, almost 700 incidents with wild boars were reported to the security services (Regional Security Centre, Police, Municipal Guard, Municipal Office,), i.e., one-third more than the number of incidents identified from crowdsourced data. The scale of these reports by months is shown in Figure 9.

From the graph it can be seen that there are discrepancies in the number of incidents, but the scale of the reports maintains a similar proportion. The total number of reported interventions is higher than the number of wild boar incidents obtained from crowd-sourcing. Not every incident was photographed by the crowd and uploaded to the social network. The observations in the social network occurred when problems with wild boars exceeded the usual levels in the city. However, the cases from the network overlap in three-quarters of the incidents with the reports of official notifications, taking into account the location of the incident. The analysis of the reports showed that interventions most often took place in city centers and in housing estates with high intensity of development. It was noted that these areas overlap with the location of areas from the crowdsourcing data.

Wildlife enthusiasts generated more information about wild boar incidents during the month of May 2020 than the statistics for reporting to the relevant services. It was also observed that the level of interest in wild boar decreases faster in the social media environment than the level of intervention.

There was a full overlap of crowdsourcing data with data from intervention reports regarding socio-spatial conflicts with rank 3, i.e., incidents posing the highest risk. By comparison, conflicts with rank 1 outweigh the reports. This shows that there is high coverage for these most important incidents.

## 4. Discussion

The local authorities should rely on the most valuable and up-to-date information to resolve complex problems in urban areas. In this process, information should be synthesized from various sources, not only official reports and public surveillance systems. Not all municipalities can afford to install and maintain surveillance systems covering the entire city. The study demonstrated that data crowdsourced directly from local users of social media are a valuable source of information. Crowdsourcing is a continuous process that supports observations of various phenomena over time. The Information Age is characterized by increasing volumes of data generated by users in virtual networks [42]. There are numerous opportunities for utilizing the enormous quantities of readily available digital data [36,43]. City residents generate information that is geotagged with specific locations where community members live and spend their free time. Social media users are often the first observers of complex urban processes and the first creators of the relevant digital content.

The crowdsourced social media data analyzed in this study were rich in content relating to socio-spatial conflicts between humans and wild boars in the city of Olsztyn. The resulting threats were assessed by analyzing a vast collection of multimedia files (606 files) generated by more than 200 local inhabitants. These resources were used to determine the type and location of threats, and the frequency of wild boar incidents in the city. It should be noted that the multimedia repository was created within a relatively short period of time (7–8 months), and effectively illustrates the scale of socio-spatial conflicts over time. The frequency with which multimedia files were uploaded to social networking sites (two files per day) was sufficient to observe certain regularities. Most multimedia files were uploaded in April, May, and June, which could be related to lockdown restrictions in public spaces during the COVID-19 pandemic. It should also be noted that the creators of multimedia files did not perceive wild boars as a cause of socio-spatial conflicts, but more as an attraction to defeat boredom during lockdown. Most content creators were motivated by their fascination with wildlife. The COVID-19 pandemic enhanced people’s sensitivity to atypical events in urban space and increased their activity on social media. The users presented information that could be used in research, but without their involvement [33,44]. This requirement has to be met for the analyzed photographs to be objective and reliable sources of data.

Crowdsourced data were characterized by satisfactory resolution, and clear images of objects and the surroundings, which supported the determination of the severity of socio-spatial conflict involving wild boars with the use of the proposed methodology. Research studies of this type have never been published in the literature, although the photointerpretation of multimedia files is a method that has been previously applied to identify other phenomena in urban space [45,46]. Despite the above, the analyzed crowdsourced data have certain limitations. One of these is the abundance of data, which have to be filtered to select resources that are repeatable, geotagged, have high resolution, and have been generated by reliable content creators. Not all of the analyzed crowdsourced data were sufficiently geotagged to support accurate determinations of the type and severity of conflicts resulting from the occurrence of wild boars in city space. Based on a preliminary analysis of 606 files, only 212 locations could be identified, and they accounted for around 50% of the locations presented in multimedia files. Geographical identification data in the evaluated photographs were highly general, and they supported the identification of city districts, such as Kusociński Park and the Old Town. These districts cover a large area; therefore, the process of localizing wild boars could not be fully automated. Photointerpretation requires a working knowledge of a city to narrow down the identified locations to specific address points. The second limitation was that crowdsourced data did not cover the entire area of the city. Photographs depicting wild boars were not available in locations where media users did not live or which were visited less frequently due to COVID-19 restrictions. There are many recreational areas within the administrative boundaries of Olsztyn, including lakes, parks, and the Municipal Forest. Traces of wild boar activity have been identified in these areas, which indicates that the absence of multimedia files relating to these parts of the city does not rule out potential threats. However, the absence of direct human–wild boar interactions decreases the rank of these potential threats. The third limitation was that not all types of threats could be effectively identified. Not all categories of socio-spatial conflicts involving wild boars could be determined based on the analyzed multimedia files. Secondary threats, including the transmission of parasites and disease, were particularly difficult to identify, and the psychological risks associated with the presence of wild boars near adults and children were assessed only to a limited extent.

## 5. Conclusions

The results of this study confirmed the research hypothesis that crowdsourced multimedia data generated by local users of social media can be supporting material, sufficient to determine the location, severity, and distribution (density) of conflict situations resulting from the encroachment of wild boars into urban areas. The severity and distribution of conflicts were determined with the use of visual materials (photographs) that are widely published by users in social networking sites. The proposed innovative method for determining the severity of socio-spatial conflicts involving wild boars in urban space can also be applied to identify and evaluate other recurring phenomena in cities. The described method is easy to implement, and accounts for the following criteria: frequency of wild boar incidents, incident locations, and the severity (rank) of the resulting threats. The information about various types of socio-spatial conflicts in the city can be used by the local authorities to resolve and alleviate these problems; for example, by developing long-term plans for managing the wild boar population [47,48,49].

In analyses of specific phenomena in urban space, data crowdsourced from social media can be an equally valuable source of information to data that are collected with the use of conventional methods (urban surveillance, surveys, incidents reported by local community members to municipal security services). This was confirmed by validating the data with data from security service intervention reports. The weakness of the approach is the incomplete coverage of the crowdsourced data of the city space. Data is usually produced by enthusiasts who are not always in every part of the city. In addition, there is a significant repetition of images and a multitude of shots, so these resources must be filtered by eliminating files that did not contain images of wild boars; there are also shots of wild boars originating from locations other than the monitoring city. Therefore, the crowdsourced data can be regarded as a supplementary resource in research. The greatest advantage of crowdsourced data is that they are up-to-date and are posted on social media immediately after a given phenomenon has been sighted by the users.

In conclusion, this study demonstrated that crowdsourced social media data are highly useful. They are a potentially valuable source of information about the type and severity of socio-spatial conflicts. It should be noted that even the most efficient urban administrators lack the necessary human and financial resources to regularly monitor human–wild boar conflicts. The results of this study confirm that crowdsourced social media data offer a quick and cost-effective alternative to traditional data collection methods. The present findings are consistent with the results of other studies, which demonstrated that social media are useful sources of information for social and environmental monitoring in areas where reliable data are scarce [15,50,51].

## Figures and Tables

**Table 1:** Classification of the threats associated with the number and location of wild boar incidents in Olsztyn.

No.	Nuisance Category	Nuisance Scale		
		Low	Medium	High
1.	Location of wild boar incidents in Olsztyn	City periphery, other locations.	City periphery and other locations that have elements of housing estates.	City center, housing estates.
2.	Number of wild boar threats reported monthly in Olsztyn	1–10	11–20	more than 20

Source: own elaboration.

**Table 2:** Categories of socio-spatial conflicts involving wild boars in Olsztyn.

No.	Category of Threat	Type of Threat	Characteristics	Rank
1	Damage to property	foraging (digging, rooting) (I)	destruction of: - lawns - flower beds - gardens, shrubs, and parks	1 3 3
		nesting (contamination and noise) (II)	destruction of: - lawns - flower beds - gardens, shrubs, and parks	1 2 3
		spread of garbage (III)	- foraging in rubbish bins - natural habitat (excrement)	2 1
2	Safety risks:	wild boars cross public roads and forage on road lanes (IV)	-	3
		wild boars cause traffic accidents (V);	-	3
		wild boars attack humans (Va)	-	3
3	Psychological risks	occurrence of wild boars (VI, VII)	- near adult people; - near children	2 3
		other behaviors, including fighting between males, copulation, sows with offspring (VIII)	-	3
4	Secondary risks	transmission of parasites and disease (IX)	-	2

Source: the authors.

**Table 3:** Crowdsourced data on wild boars in Olsztyn.

Nazwa Grupy	Private Group *“Dziki w Olsztynie”* (“Wild Boars in Olsztyn”)	Website 1 *“Olsztyn Kocha Dziki”* (“Olsztyn Loves Wild Boars”)	Website 2 *“Gdzie są Dziki Olsztyn”* (“Where are the Wild Boards Olsztyn”)	Website 3 *“Dziki w Olsztynie”* (“Wild Boars in Olsztyn”)
number of participants	3357	326	103	214
the date of group establishment	26 March 2020	17 June 2019	6 July 2020	6 March 2020
the number of entries	382	35	10	3
the number of active participants (entry authors)	229	-	8	2
the number of multimedia files (including photographs and videos)	616	61	9	3
the aim of the establishment	wild boar enthusiasts	wild boar enthusiasts	providing information	providing information and issuing warnings

Source: own elaboration.

**Table 4:** Results of the photointerpretation-based analysis of crowdsourced multimedia files.

Indicators	Quantitative Data							
Period	March 2020	April 2020	May 2020	June 2020	July 2020	August 2020	September 2020	October 2020
Number of hazards	4	32	230	149	127	32	17	16
Number of wild boar threats in the city of Olsztyn according to the scale in Table 1	1	3	3	3	3	3	2	2
Number of rank 2 and 3 hazards	1	17	148	91	77	17	12	12
Threat assessment: location of wild boars in the city of Olsztyn according to the scale in Table 2	2	3	3	3	3	3	3	3
Severity of socio-spatial conflicts involving wild boars in the city of Olsztyn	low	high	high	high	high	high	medium	medium

Source: own elaboration.

## Data Availability

Data was obtained from Municipal Wardens in Olsztyn. The data is on the authors’ resources.
